# Intrahepatic *Klebsiella pneumoniae* is associated with host cellular dysfunction in metabolic dysfunction-associated steatohepatitis

**DOI:** 10.1128/spectrum.00981-26

**Published:** 2026-08-04

**Authors:** Seon Yeong Park, Kyung Eun Kim, Hyeong Seok An, Jung Wook Yang, Yun Hak Kim, Gu Seob Roh

**Affiliations:** 1 Department of Convergence Medical Sciences, Pusan National University 34996, Yangsan, Republic of Korea; 2 Department of Anatomy, College of Medicine, Metabolic dysfunction liver disease Research Center, Institute of Medical Science, Gyeongsang National University 65442, Jinju, Republic of Korea; 3 Department of Pathology, College of Medicine, Gyeongsang National University 65442, Jinju, Republic of Korea; 4 Research Institute for Convergence of Biomedical Science and Technology, Pusan National University Yangsan Hospital 194197, Yangsan, Republic of Korea; 5 Department of Anatomy, Pusan National University 34996, Yangsan, Republic of Korea; 6 Department of Biomedical Informatics, Pusan National University 34996, Yangsan, Republic of Korea; City of Hope Department of Pathology, Duarte, California, USA

**Keywords:** liver diseases, microbiota, *Klebsiella pneumoniae*

## LETTER

**Fig 1:**
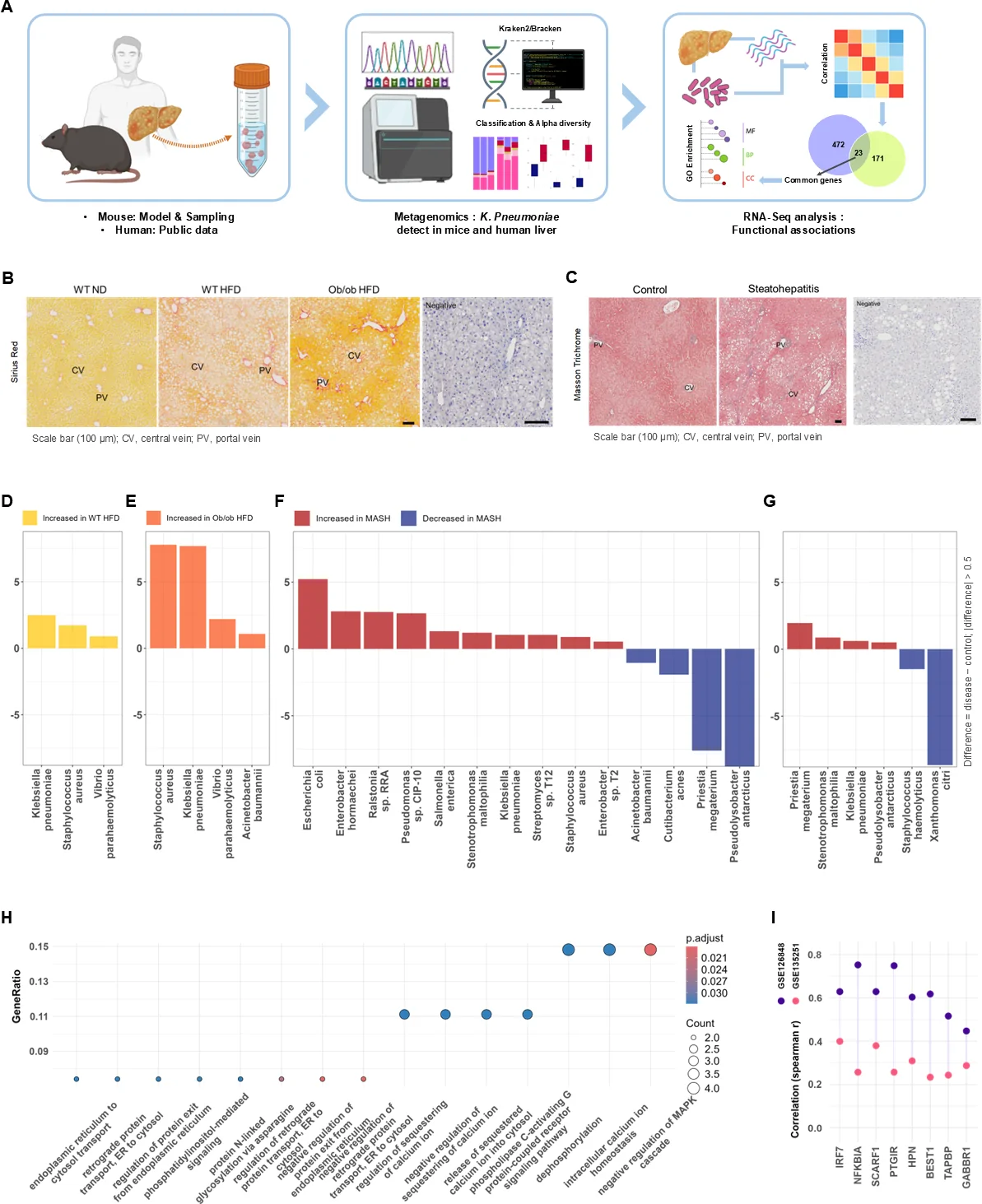
Intrahepatic microbial alterations and *K. pneumoniae*-associated host responses in MASH. (**A**) Study design showing integration of murine and human liver samples, microbial profiling, and host–microbe correlation analyses. Murine liver samples included ND-fed WT mice (*n* = 3), HFD-fed WT mice (*n* = 3), and HFD-fed ob/ob mice (*n* = 3). Two human liver RNA-seq data sets were obtained from NCBI: GSE126848 (MASH, *n* = 15; controls, *n* = 14) and GSE135251 (MASH, *n* = 53; controls, *n* = 10). (**B**) Sirius Red staining demonstrates increased hepatic collagen deposition and fibrosis in HFD-fed WT and ob/ob mice compared with ND-fed WT mice; the negative control is unstained. (**C**) Masson trichrome shows increased fibrosis in human steatohepatitis versus controls; the negative control is unstained. (**D–G**) Relative abundance of bacterial species in intrahepatic tissues: (**D**) HFD-fed WT versus ND-fed WT mice (GSE167264), (**E**) HFD-fed ob/ob versus ND-fed WT mice (GSE167264), (**F**) MASH versus controls (GSE126848), and (**G**) MASH versus controls (GSE135251). Bars indicate differences in mean relative abundance between disease and control groups (disease – control). Species with an absolute difference of >0.5 are shown for visualization. (**H**) GO enrichment of host genes positively correlated with intrahepatic *K. pneumoniae* (overlap of GSE126848 and GSE135251; adjusted *P* < 0.05). (**I**) Spearman correlations between *K. pneumoniae* abundance and inflammation-related genes; eight genes show positive correlations in both cohorts (*P* < 0.1).

Metabolic dysfunction-associated steatohepatitis (MASH), defined by hepatic steatosis, lobular inflammation, and fibrosis, is a major risk factor for cirrhosis and hepatocellular carcinoma (1–3). Increasing evidence implicates the gut–liver axis and microbiota dysbiosis as key drivers of MASH pathogenesis (4). A notable example is high-alcohol-producing *Klebsiella pneumoniae*, which induces hepatic steatosis and inflammation in animal models via endogenous ethanol production (5). *K. pneumoniae* is also an important pathogen linked to hospital-acquired infections and pyogenic liver abscesses (6). However, most studies have focused on indirect gut-derived effects, with limited attention to microbial communities residing within the liver (intrahepatic microbiota). Here, we investigated intrahepatic microbial alterations in MASH using metatranscriptomic analysis of murine liver tissues and publicly available human liver RNA-seq data sets (Fig. 1A).

Four-week-old male wild-type (WT) and leptin-deficient ob/ob mice were fed either a high-fat diet (HFD) or normal diet (ND) for 20 weeks to induce experimental MASH. Human liver sections from control and steatohepatitis patients with cholangiocarcinoma were obtained from the Department of Pathology, College of Medicine, Gyeongsang National University (Jinju, Republic of Korea). Histological analysis demonstrated increased fibrosis-associated collagen deposition in HFD-fed ob/ob mice (Fig. 1B) and increased hepatic fibrosis in human steatohepatitis samples compared with controls (Fig. 1C), consistent with the key pathological features of MASH (7, 8). Liver tissues from mice and human cohorts (GSE126848 and GSE135251) were analyzed using a metatranscriptomic workflow, in which host-unmapped RNA-seq reads were classified with Kraken2 and abundance estimates were generated using Bracken.

In murine models, species-level relative abundances derived from Bracken outputs were visualized as the mean differences in relative abundance between disease and control groups (disease – control). These descriptive comparisons showed higher mean relative abundance of *K. pneumoniae* and *Staphylococcus aureus* in HFD-fed WT and ob/ob mice compared with ND-fed WT mice (Fig. 1D and E). Consistent trends were observed in human data sets, where *K. pneumoniae* repeatedly showed higher mean relative abundance in MASH samples compared with controls (Fig. 1F and G). In supplementary ANCOM-BC analysis, *K. pneumoniae* reached FDR-adjusted significance in GSE126848 (log fold change = 4.33, *q* = 4.25 × 10^−7^) and showed the same positive direction in GSE135251 (log fold change = 0.780, nominal *P* = 0.0027; *q* = 0.098) (Fig. S1A and B). The repeated enrichment of *K. pneumoniae* across cohorts supports its potential relevance in MASH. We next assessed the overall microbial diversity (Fig. S1C through F). Alpha diversity (observed richness, Shannon, and Simpson indices) was calculated in R (phyloseq/vegan) and compared between groups using the Wilcoxon rank-sum test. Alpha diversity metrics did not differ significantly between MASH and controls, but all data sets showed a consistent trend toward higher diversity in MASH.

To investigate host responses associated with intrahepatic microbes, gene-level host RNA-seq counts from the two human cohorts were first processed using edgeR, and differentially expressed genes between MASH and control groups were defined using a nominal *P* value < 0.05. Microbial relative abundance and host gene expression values were normalized prior to correlation analysis, and Pearson correlation testing was performed between intrahepatic *K. pneumoniae* abundance and host gene expression. Positively correlated genes with *P* < 0.05 were retained and subjected to gene ontology enrichment analysis in each cohort. Enriched pathways included negative regulation of mitogen-activated protein kinase (MAPK) signaling, intracellular calcium ion homeostasis, protein dephosphorylation, and G protein-coupled receptor–phospholipase C signaling (Fig. 1H), suggesting the potential involvement of MAPK regulatory responses within inflammatory and cellular stress-related contexts rather than direct activation of inflammatory signaling (9).

For inflammation-related genes, associations with intrahepatic *K. pneumoniae* abundance were further evaluated using Spearman correlation. Candidate genes showing consistent positive associations with *K. pneumoniae* abundance at an exploratory threshold of *P* < 0.1 in both independent human cohorts—including *IRF7*, *NFKBIA*, *SCARF1*, *PTGIR*, *HPN*, *BEST1*, *TAPBP*, and *GABBR1*—were highlighted (Fig. 1I).

Together, these findings indicate that intrahepatic microbial alterations, particularly the enrichment of *K. pneumoniae*, are associated with inflammatory and metabolic stress pathways in MASH. Although low-biomass microbial profiling is susceptible to technical contamination, the reproducible, group-associated enrichment patterns observed across independent data sets are less consistent with contamination and more likely reflect biological variation within the hepatic microenvironment. Future studies incorporating spatially resolved microbial detection, longitudinal clinical cohorts, and mechanistic experimental models will be important to further clarify the relationship between intrahepatic *K. pneumoniae* enrichment and host stress-related responses in MASH.

In conclusion, our study highlights intrahepatic microbiota as a potentially important component of MASH pathobiology and identifies *K. pneumoniae* as a recurrent intrahepatic species associated with host stress-related responses. These findings provide a rationale for further investigation into microbiome-based biomarkers and the development of antimicrobial or microbiome-modulating therapeutic strategies. In addition, validation in larger patient cohorts may help determine whether intrahepatic microbial signatures could serve as clinically relevant biomarkers for disease stratification or therapeutic response prediction.

## Data Availability

The data sets analyzed during the current study are available in the NCBI Gene Expression Omnibus (GEO) repository, GSE167264, GSE126848, and GSE135251. All other data supporting the findings of this study are available from the corresponding author upon reasonable request.

## SUPPLEMENTAL MATERIAL

Fig. S1 spectrum.00981-26-s0001.TIF ANCOM-BC differential abundance analysis and alpha diversity comparison between MASH and healthy controls.

Supplemental text spectrum.00981-26-s0002.docx Legend for Fig. S1 and additional experimental details.

Table S1 spectrum.00981-26-s0003.pdf Clinical characteristics for two human cohorts analyzed in this study.

ASM does not own the copyrights to Supplemental Material that may be linked to, or accessed through, an article. The authors have granted ASM a non-exclusive, world-wide license to publish the Supplemental Material files. Please contact the corresponding author directly for reuse.
