# Supplementary material for: Intrahepatic Klebsiella pneumoniae is associated with host cellular dysfunction in metabolic dysfunction-associated steatohepatitis
Source: Microbiol Spectr. 2026 Aug 4;14(9):e00981-26. doi: 10.1128/spectrum.00981-26 (PMC13532251; doi:10.1128/spectrum.00981-26)
Supplement: Supplementary file 1 [file spectrum.00981-26-s0003.pdf]

Supplementary Table 1. Clinical characteristics for two human cohorts analyzed in this study. Demographic, anthropometric, biochemical, and liver histology features are summarized as reported in the original datasets.

Dataset 1: GSE126848

Category	Feature	Normal-weight controls (n = 14)	Obese controls (n = 12)	MASLD (n = 15)	MASH (n = 16)
Demographics	Age, years (mean ± SD)	39.5 ± 12.0	36.6 ± 10.2	39.4 ± 10.6	38.9 ± 17.0
	Male sex (%)	100	100	60	75
Anthropometrics	BMI, kg/m² (mean ± SD)	23.1 ± 1.6	33.2 ± 1.3	32.8 ± 5.1	33.9 ± 6.2
Liver biochemistry	ALT, U/L (mean ± SD)	31.8 ± 8.9	39.7 ± 15.8	96.7 ± 55.8	115.0 ± 50.0
	AST, U/L (mean ± SD)	33.4 ± 9.0	41.2 ± 15.4	45.1 ± 23.8	54.8 ± 20.6
Histology	Steatosis ≥ grade 2 (%)	0	8.3	66.7	87.5
	Lobular inflammation present (%)	0	0	86.7	100
	Hepatocellular ballooning present (%)	0	0	0	62.5
	Fibrosis ≥ stage 1 (%)	0	0	6.7	81.3

Values are presented as mean ± standard deviation or percentage, as indicated.

ALT, alanine aminotransferase; AST, aspartate aminotransferase; BMI, body mass index; MASLD, metabolic dysfunction–associated steatotic liver disease; MASH, metabolic dysfunction–associated steatohepatitis.

Dataset 2: GSE135251

**Category**	**Feature**	**MASH**
Demographics	Number of patients	141
	Age, years (mean ± SD)	52.6 ± 12.4
	Male sex, n (%)	87 (61.7)
Anthropometrics	BMI, kg/m² (mean ± SD)	30.8 ± 5.0
Metabolic comorbidities	Type 2 diabetes, n (%)	64 (45.4)
	HbA1c, mmol/mol (mean ± SD)	45.8 ± 12.2
Liver biochemistry	ALT, U/L (mean ± SD)	65.3 ± 41.7
	AST, U/L (mean ± SD)	40.7 ± 20.4
	Platelet count, $\times 10^9$/L (mean ± SD)	237.8 ± 66.0
Histological features	Steatosis grade ≥2, n (%)	97 (68.8)
	Hepatocellular ballooning present, n (%)	99 (70.2)
	Lobular inflammation present, n (%)	125 (88.7)
	Fibrosis stage ≥1, n (%)	71 (50.4)
Disease classification	Diagnosis of MASH, n (%)	98 (69.5)
	NAS score ≥4, n (%)	94 (66.7)
	SAF activity score ≥2, n (%)	97 (68.8)

Values are presented as mean ± standard deviation or number (percentage), as indicated.

ALT, alanine aminotransferase; AST, aspartate aminotransferase; BMI, body mass index; MASH, metabolic dysfunction–associated steatohepatitis; NAS, NAFLD activity score; SAF, steatosis–activity–fibrosis.
