# Supplementary material for: Intrahepatic Klebsiella pneumoniae is associated with host cellular dysfunction in metabolic dysfunction-associated steatohepatitis
Source: Microbiol Spectr. 2026 Aug 4;14(9):e00981-26. doi: 10.1128/spectrum.00981-26 (PMC13532251; doi:10.1128/spectrum.00981-26)
Supplement: Supplementary file 2 [file spectrum.00981-26-s0002.docx]

**Supplementary Figure 1. ANCOM-BC differential abundance analysis and alpha diversity comparison between MASH and healthy controls.**

(A and B) ANCOM-BC analysis of Bracken-derived species-level count matrices from (A) GSE126848 and (B) GSE135251. Bars indicate log fold changes, with positive values representing enrichment in MASH and negative values representing enrichment in healthy controls. For visualization, taxa with nominal p<0.05 were ranked by absolute log fold change, and the top species are shown. Asterisks indicate nominal p-value levels: *p<0.05, **p<0.01, and ***p<0.001. K. pneumoniae is highlighted with a star. FDR-adjusted q values were used for formal differential abundance assessment. (C) WT HFD mouse model, (D) ob/ob HFD mouse model, (E) GSE126848, and (F) GSE135251. Shannon diversity, Simpson index, and observed species richness are compared between MASH and control groups. Statistical significance was assessed by the Wilcoxon rank-sum test.

**Material and methods**

**Experimental animals**

Four-week-old male C57BL/6J wild-type (WT) and leptin-deficient ob/ob mice were purchased from Central Laboratory Animal, Inc. (Seoul, Republic of Korea). For an HFD (60 % kcal fat, Research Diets, New Brunswick, NJ, USA)-fed MASH mouse models, WT and ob/ob mice of at least 5 weeks of age were fed an HFD or normal diet (ND, 5 % kcal fat, Harlan Laboratories, Inc., Indianapolis, IN, USA) for 20 weeks. All mice were housed in virus-free facilities on a 12-h light/12-h dark cycle. All animal experiments were performed in accordance with the National Institutes of Health Guidelines on the Use of Laboratory Animals. The Animal Care Committee for Animal Research of Gyeongsang National University approved the study protocol (GNU-190701-M0033).

**Human tissues**

Human liver sections from control and steatohepatitis from patients with cholangiocarcinoma were provided by the Department of Pathology, College of Medicine, Gyeongsang National University (Jinju, Republic of Korea). All subjects provided written informed consent. The study also conformed to the Declaration of Helsinki and Declaration of Istanbul and obtained approval from the Institutional Review Board of the Gyeongsang National University Hospital (GNUH IRB 2025-09-003).

**Tissue collection and histological analysis**

For histological analysis, mice were anesthetized and perfused with 4% paraformaldehyde in 0.1 M phosphate buffered saline. Livers were fixed in 4% paraformaldehyde for 12 h at 4°C, embedded in paraffin, and cut into 5-μm sections. Histological fibrosis was examined by Picro-Sirius Red (Sigma-Aldrich, Inc., St Louis, MO, USA) and Masson trichrome (MT, Polysciences Inc., PA, USA) staining according to the manufacturer’s protocols. The sections were visualized using a microscope slide scanner (Motic, Hong Kong, China).

**Public RNA-seq datasets**
Two liver RNA-seq datasets were obtained from NCBI: GSE126848 (MASH, n=15; controls, n=14) and GSE135251 (MASH, n=53; controls, n=10). Clinical metadata are provided in Table S1.

**Read preprocessing, host mapping, and microbiome profiling**

Paired-end reads were quality-filtered and adapter-trimmed (FastQC/Trimmomatic), aligned to the human genome (Bowtie2), and unmapped reads were extracted for metatranscriptomic profiling. Unmapped reads (≥100 nt) were classified using Kraken2 (v2.1.1; default settings; standard database), and species-level abundances were estimated with Bracken.

**Alpha diversity & relative abundance**

Alpha diversity (observed richness, Shannon, and Simpson indices) was calculated in R (phyloseq/vegan) and compared between groups using the Wilcoxon rank-sum test. Species-level relative abundances were derived from Bracken outputs and visualized as differences in mean relative abundance between disease and control groups (disease – control).

**Differential expression analysis**

Differentially expressed genes (DEGs) were identified using two human RNA-seq datasets retrieved from the NCBI SRA. Gene-level counts were processed with the edgeR package, and statistical testing was performed to compare MASH and control groups. Genes with a nominal p-value < 0.05 were considered significantly differentially expressed and were used for subsequent analyses.

**Correlation analysis**

Microbial relative abundance was integrated with host gene expression, and both were normalized prior to correlation testing. Pearson correlations were computed, and positively correlated genes (p<0.05) were retained for downstream analyses. Associations between intrahepatic K. pneumoniae abundance and selected inflammation-related genes were assessed using Spearman correlation; genes with p<0.1 in both cohorts were highlighted.

**Gene ontology (GO) enrichment analysis**

GO enrichment analysis was performed on positively correlated DEGs to assess their functional relevance in MASH. Analyses were conducted independently for the human datasets. The Biological Process ontology was tested using the following parameters: keyType="ENSEMBL", minGSSize=10, maxGSSize=500, pvalueCutoff=0.05, qvalueCutoff=0.05.

**ANCOM-BC analysis**

Differential abundance analysis of microbial species was performed using ANCOM-BC on Bracken-derived species-level count matrices. Analyses were conducted independently for the two human datasets, GSE126848 and GSE135251. Disease group was used as the variable of interest, with healthy controls set as the reference group and MASH as the comparison group. The model was specified as formula="Group", and p values were adjusted for multiple testing using the Benjamini–Hochberg method. To retain low-prevalence taxa potentially showing disease-associated detection patterns, no prevalence filtering was applied (prv_cut=0). Samples with library sizes below 1,000 microbial reads were excluded (lib_cut=1000). Structural-zero detection was disabled (struc_zero=FALSE) to evaluate differential abundance based on the standard ANCOM-BC model rather than group-specific absence/presence classification. The analysis was performed with conserve=TRUE, alpha=0.05, and global=FALSE. Taxa with FDR-adjusted q values <0.05 were considered differentially abundant.
